# Molecular analysis of human papillomavirus in never-smokers with non-small cell lung cancer

**DOI:** 10.3892/ol.2014.2713

**Published:** 2014-11-19

**Authors:** SHUN-ICHI ISA, YU KURAHARA, SATOMI YAMAMOTO, AKIHIRO TAMIYA, NAOKI OMACHI, KAZUHIRO ASAMI, KYOICHI OKISHIO, TOMOKI UTSUMI, NORIMASA ITO, HYUNG-EUN YOON, AKIHIDE MATSUMURA, SHINJI ATAGI, TOMOYA KAWAGUCHI

**Affiliations:** 1 Clinical Research Center, Sakai, Japan; 2 Division of Internal Medicine, National Hospital Organization Kinki-Chuo Chest Medical Center, Sakai, Japan; 3 Division of Chest Surgery, National Hospital Organization Kinki-Chuo Chest Medical Center, Sakai, Japan; 4 Department of Respiratory Medicine, Graduate School of Medicine, Osaka City University, Osaka, Japan

**Keywords:** lung cancer, human papillomavirus, never-smokers, prospective registry

## Abstract

The causes of lung cancer in never-smokers remain unclear. The potential contribution of human papillomavirus (HPV) to the carcinogenesis of non-small cell lung cancer (NSCLC) has been reported. In 2008, a prospective registry of never-smokers with NSCLC was established at the Kinki-Chuo Chest Medical Center, Sakai, Osaka, Japan. Never-smokers with NSCLC were consecutively enrolled onto the registry. Of these patients, 114 with large tumor specimens, the majority of which were surgical tissues, were selected. In total, 23 of the most clinically relevant HPV types were assayed using polymerase chain reaction amplification of the viral genome. Following exclusion of samples with suboptimal quality, DNA was extracted from 96 formalin-fixed paraffin-embedded samples. These 96 cases consisted of 82 females (85.4%) and 14 males (14.6%), with a median age of 67 years (range, 29–83). Almost all cases (93.8%) were of the adenocarcinoma histological subtype. Despite confirmation of the quality and amount of DNA, HPV type 6 was detected in only one case (1.1%). Furthermore, no other samples examined were positive for any other HPV types. The results therefore suggest that HPV does not play a major role as the driving oncogenic event in never-smokers with NSCLC.

## Introduction

While lung cancer is the leading cause of cancer-related mortality and is predominantly caused by tobacco smoke, ~25% of all lung cancers worldwide are not attributable to tobacco smoke (1). In fact, in a large Japanese cohort study of >20,000 patients with non-small cell lung cancer (NSCLC), 30% were never-smokers (2). Lung cancer in never-smokers is unique with regard to its clinical characteristics and is suggested to be a distinct disease, which has emerged as a global public health concern (3,4). Investigations into the molecular mechanisms responsible for the disease are urgently necessary in order to improve therapeutic strategies. A number of etiological factors have been proposed, with infection with oncogenic human papillomavirus (HPV) as one of these (5–7). A recent study on the comprehensive genome sequencing of cancer revealed that the apobec3b protein was associated with carcinogenesis in multiple cancers, including lung cancer (8). Viral infection is known to play a significant role in upregulating proteins. In fact, cervical cancer and pharyngo-laryngeal cancer have been classified into a unique mutational signature that is associated with HPV (8).a

A meta-analysis previously demonstrated that the prevalence of HPV in lung cancer is highly variable around the world, with higher HPV frequencies observed in East Asian countries compared with European countries (9). However, inadequate sample collection or handling errors leading to contamination may contribute to these variations (10). At the Kinki-Chuo Chest Medical Center (Sakai, Osaka, Japan) only patients with lung diseases are treated, which is an advantage in the avoidance of clinical sample contamination. In the present study, a prospective registry investigation into the involvement of HPV in NSCLC in never-smokers at the Kinki-Chuo Chest Medical Center is reported.

## Patients and methods

### Tissue specimens from prospective registry of never-smokers

In 2008, a prospective registry of never-smokers with NSCLC was established at the National Hospital Organization Kinki-Chuo Chest Medical Center. Subsequent to obtaining written informed consent, patients were enrolled prospectively and consecutively to determine if they had newly diagnosed or histologically or cytologically confirmed NSCLC. The patients were never-smokers (<100 cigarettes/lifetime) and had tumor tissue samples available for analysis. All patients were requested to complete a detailed questionnaire, including confirmation of never-smoker status and any family history of cancer and passive smoking status (11). A total of 250 patients were enrolled between March 2008 and January 2010, and clinical data was collected from medical records and questionnaires. Among the 250 patients, 114 with sufficiently large tumor specimens were selected, the majority of which were surgical tissues.

This study was approved by the Institutional Review Board of the National Hospital Organization Kinki-Chuo Chest Medical Center.

### HPV DNA genotyping

HPV genotyping was performed using a polymerase chain reaction (PCR)-based microarray system (GeneSQUARE, Kurabo, Osaka, Japan) to detect 23 HPV types, including high-risk (HPV types 16, 18, 31, 33, 35, 39, 45, 51, 52, 56, 58, 59 and 68), low-risk or risk-unknown types (HPV types 6, 11, 30, 34, 40, 42, 53, 54, 61 and 66). This system uses type-specific primers and probes that are designed to detect the E2, E6, E7, L1 and L2 genes. The target HPV-DNA was amplified by PCR using a Mastercycler gradient (Eppendorf, Hambrug, Germany). PCR was performed in a total volume of 20 μl, containing multiple primers for each of the HPV types (GenBank accession numbers: AF092932, M14119, NC_001526, X05015, X74474, J04353, M12732, X74476, M74117, M62849, X74478, M73236, X74479, M62877, X74481, X74482, U37488, X74483, D90400, X77858, U31793, U31794, M73258 and 123996136 for HPV types 16, 18, 31, 33, 35, 39, 45, 51, 52, 56, 58, 59 and 68, respectively) FastStart Taq DNA polymerase (Roche Diagnostics, Basel, Switzerland), dNTP mix, dUTP labeled with Cy3 and specimen DNA. A total of 100–1,000 ng/3 μl specimen DNA was used in each reaction. PCR conditions were as folows: 4 min at 95°C, followed by 40 cycles of 95°C for 30 sec, 65°C for 30 sec, 72°C for 1 min, and one cycle of 72°C for 7 min. The DNA samples were then applied to HPV genotyping arrays and hybridization was performed at 65°C for 1 h. Samples were washed and dried, and analysis was performed using a micro-array scanner (12). The HPV positivity rate was calculated based on positive results by either consensus PCR or genotyping, both of which use different primer settings. Molecular analysis was performed at least twice for confirmation.

## Results

### Clinical characteristics

Following the exclusion of the samples with suboptimal quality from the 114 patients, DNA was successfully extracted from 96 formalin-fixed paraffin-embedded surgical tissues. The clinical characteristics of these 96 cases are summarized in Table I. The cases consisted of 82 female patients (85.4%) and 14 male patients (14.6%), with a median age of 67 years (range, 29–83 years). The majority of the cases exhibited adenocarcinoma histology (93 patients; 93.8%). Molecular biological analysis of the 93 patients with adenocarcinoma showed 59 epidermal growth factor receptor *(EGFR)* mutations (63.4%), five *KRAS* mutations (5.4%) and four *EML4-ALK* rearrangements (4.3%).

### HPV detection

HPV type 6 was detected in only one case (1.0%) among the 96 tumor samples. The patient was a 49-year-old female who presented with an *EGFR* mutation (exon 19) with wild-type *KRAS* and *ALK*. The patient had previously undergone surgery twice for condyloma acuminatum, and had stage I adenocarcinoma. During a 25-year period, the patient was subjected to environmental tobacco smoke exposure (from 28 to 53 years of age at home, and from 58 to 59 years at work). At five years post-surgery, the patient experienced a recurrence of the lung cancer. Following treatment with gefitinib (250 mg/day) for one year, the patient succumbed to brain metastasis subsequent to several rounds of chemotherapy.

No other patients examined were positive for any HPV types, including high-risk (HPV types 16, 18, 31, 33, 35, 39, 45, 51, 52, 56, 58, 59 and 68), low-risk or risk-unknown types (HPV types 6, 11, 30, 34, 40, 42, 53, 54, 61 and 66).

## Discussion

In the present study, a low-risk HPV type was evident in one case among 96. However, no HPV of any type was detected in any other tumors. These results suggest that HPV does not play a major role as the driving oncogenic event in lung cancer in never-smokers in Osaka, Japan.

It is known that HPV types essentially cause all human cervical cancers (13). During the last two decades, several studies have examined the possible involvement of HPV in non-genital cancers and have proposed the presence of HPV in esophageal, laryngeal, oropharyngeal, urothelial, breast, colon and lung cancer (14). The virus is able to infect the oral mucosa, the larynx and the bronchial tissues, which therefore may be the primary source of HPV detected in the lung (15). In fact, Carpagnano *et al* demonstrated the presence of HPV in the exhaled breath condensate of lung cancer patients (16).

Although the data from the present study suggest that HPV involvement in lung cancer is unlikely in never-smokers, the issue remains controversial. According to previous studies, the prevalence of HPV is not uniform (9,17). Regional differences exist within Asia, the Americas, Europe and throughout Japan. Higher levels are observed in Okinawa and southern areas of Japan compared with Tokyo and central areas (9).

There are several advantages to the present study. Firstly, the data in this study is accurate, as it was obtained by detailed questionnaires and interviews of the patients and their families. Secondly, the HPV genotyping used a high precision test and included a range of 23 HPV types, including low- and high-risk types. In order to further clarify HPV carcinogenesis, a nationwide prospective study of the viral molecular epidemiology of Japan is required. This should consider the association between viral infection and carcinogenesis, and regional differences in HPV prevalence (7).

In conclusion, a low-risk HPV type was evident in one patient sample, and no other lung cancer tumors that were examined were positive for any HPV types in the never-smokers. These results suggest that HPV does not play a major role as the driving oncogenic event in never-smokers in Osaka, central Japan.

## Figures and Tables

**Table I:** Patient characteristics among never-smokers with non-small cell lung cancer.

	Male, n (%)	Female, n (%)	Total, n (%)
Pathological stage			
I	8 (13.6)	51 (84.4)	59 (61.5)
II	2 (40.0)	3 (60.0)	5 (5.2)
III	2 (11.0)	16 (88.9)	18 (18.8)
IV	2 (14.3)	12 (85.7)	14 (14.6)
Total	14 (14.6)	82 (85.4)	96 (100)
Histology			
Adenocarcinoma	11 (12.2)	79 (87.8)	90 (93.8)
Squamous cell carcinoma	3 (75.0)	1 (25.0)	4 (4.2)
Small cell carcinoma	0 (0.0)	0 (0.0)	0 (0.0)
Others	0 (0.0)	2 (100)	2 (2.1)
EGFR mutation status			
Positive	5 (8.8)	52 (91.2)	57 (59.4)
Exon 19	1 (4.3)	22 (95.7)	23 (24.0)
L858R	3 (12.0)	22 (88.0)	25 (26.0)
Others	1 (11.1)	8 (88.9)	9 (9.4)
Negative	9 (23.1)	30 (76.9)	39 (40.6)
HPV genotyping status			
Positive	0 (0.0)	1 (100.0)	1 (1.0)
Negative	14 (14.7)	81 (85.3)	95 (99.0)

EGFR, epidermal growth factor receptor; HPV, human papillomavirus.
